# Associations between prematurity, postpartum anxiety, neonatal intensive care unit admission, and stress

**DOI:** 10.3389/fpsyt.2024.1323773

**Published:** 2024-02-23

**Authors:** Semra Worrall, Paul Christiansen, Asma Khalil, Sergio A. Silverio, Victoria Fallon

**Affiliations:** ^1^ Department of Psychology, Institute of Population Health, University of Liverpool, Liverpool, United Kingdom; ^2^ Fetal Medicine Unit, Liverpool Women’s NHS Foundation Trust, Liverpool, United Kingdom; ^3^ Fetal Medicine Unit, St George’s University Hospitals NHS Foundation Trust, London, United Kingdom; ^4^ Department of Women & Children’s Health, School of Life Course & Population Sciences, King’s College London, London, United Kingdom; ^5^ School of Psychology, Faculty of Health, Liverpool John Moores University, Liverpool, United Kingdom

**Keywords:** preterm birth, gestational age, postpartum anxiety, neonatal intensive care unit, maternal mental health, cross-sectional study

## Abstract

**Introduction:**

It is well established that a premature birth increases the likelihood of developing anxiety during the postpartum period, and that the environment of the neonatal intensive care unit (NICU) might be a contributing factor. Mothers of earlier premature infants may experience these anxieties to a higher degree compared to mothers of later premature infants. The aim of this study was to explore the association between prematurity and postpartum-specific anxiety, and the relationship between postpartum-specific anxiety and stress in the NICU.

**Materials and methods:**

Mothers (*N* = 237) of infants aged between birth and 12 months completed an online survey containing the Postpartum Specific Anxiety Scale – Research Short Form (PSAS-RSF) and the Parental Stressor Scale: Neonatal Intensive Care Unit (PSS:NICU). Structural equation modeling was used to analyze the relationship between gestational age and postpartum-specific anxiety, with one-way ANOVAs used to analyze this relationship with respect to categories of gestational age. Hierarchical regression models analyzed the relationship between postpartum-specific anxiety and stress in the NICU.

**Results:**

For the PSAS-RSF, Practical Infant Care Anxieties (*p =* 0.001), Maternal Competence and Attachment Anxieties (*p =* 0.033), and Infant Safety and Welfare Anxieties (*p =* 0.020) were significantly associated with week of gestation. Practical Infant Care and Infant Safety and Welfare Anxieties were significantly higher for mothers of late premature infants, compared to mothers of term infants (*p <* 0.001; *p =* 0.019). There were no significant between-group differences with respect to Maternal Competence and Attachment Anxieties. After controlling for potential confounders, Infant Safety and Welfare Anxieties were significantly associated with increased stress in the NICU (*p <* 0.001) as measured by the PSS:NICU.

**Conclusions:**

Our findings highlight the need for interventions for mothers with premature infants, which specifically target anxieties reflected in the PSAS-RSF, such as routine care and increasing maternal self-efficacy.

## 1 Introduction

Preterm birth has variable physical and psychological health impacts on both mother and infant, including impacts on bonding (1), and neurodevelopmental delays which can persist well into adulthood (2). The World Health Organization (WHO) outlines three categories of prematurity according to weeks of gestation: extremely (<28), very (28–<32), and moderate-to-late (32–<37) (3). Preterm birth affects approximately 11% of women worldwide (4). This number is increasing globally, with poor access to high-quality healthcare being a suggested driver, especially in low- and middle-income countries (5). Although rates are higher globally (6), in the UK, approximately one in seven infants born are admitted to the neonatal intensive care unit (NICU) (7), with length of stay being higher among those born earlier (8).

Considerable evidence suggests that giving birth prematurely significantly increases the risk of maternal mental health difficulties. Mothers of extremely premature infants have consistently poorer mental health outcomes compared to mothers who have later premature infants (9), including feelings of hypervigilance and panic, through NICU stays and to the transition home (10, 11). More broadly, maternal depression (12), posttraumatic stress disorder (13), and anxiety (14, 15) have been linked to the unexpectedness of the birth and loss of control regarding the infant’s health in the context of preterm birth. A recent systematic review estimated that, at one month after birth, prevalence rates of anxiety and posttraumatic stress were 41.9% and 39.9%, respectively, among parents with infants admitted to the neonatal unit (16).

Postpartum anxiety (PPA) is characterized by excessive and severe feelings of worry and restlessness following childbirth and can be maternally focused and/or infant-focused. PPA has been linked to negative consequences in mothers of term infants (17), including impaired mother**–**infant bonding (18) and irritability towards the infant (19). Mothers of premature infants are more likely to develop symptoms of anxiety, particularly in relation to NICU stays (14, 20). Although there is increasing research into the relationship between a premature birth and the onset of PPA (15), the use of measures designed for general adult populations persists (21, 22), despite evidence suggesting that they are inappropriate for use during the postpartum period (23).

Anxiety and stress are often considered comorbid and may be experienced simultaneously by mothers, particularly in the NICU, with levels of state anxiety being significantly associated with stress in mothers with infants in the NICU (24). Despite being treated synonymously, stress differs conceptually from anxiety and is experienced differently, including in the perinatal period. Stress is often a response to a situational factor over which an individual perceives they have no control (25). The NICU itself presents as a unique stressor, with the clinical environment, routines, social isolation, and having others care for their baby acting as significant sources of stress for mothers (26–28). As outlined by Wenzel (29), ‘*anxiety becomes problematic when it consumes a significant proportion of a woman’s time, takes away from her ability to fulfil major role responsibilities, and interferes with her ability to take care of herself*’. Parents are often advised their baby’s discharge from the NICU will coincide with their due date; many stays are longer (30). Such uncertainty may increase anxiousness (31), and mothers often suffer from the physical and psychological separation from their infant, impairing bonding (32).

Previous studies show a clear association between PPA and premature birth, and that adverse experiences of the NICU can increase symptoms of anxiety (21, 33). The consideration of postpartum-specific anxiety within the context of the NICU is important and has yet to be explored. This is important to consider in a clinical context, like care in the NICU, as areas for targeted intervention can be identified, which is not possible when using a general anxiety measure. Increasingly, the extrapolation of generalized measures to a perinatal context has been recognized as problematic (34) because items do not relate to the unique context of motherhood, and so scores may be skewed and not necessarily reflective of anxiety. For example, the item ‘*I feel rested*’ on the State Trait Anxiety Inventory (35) may produce inflated scores in a postpartum context, as lack of sleep is common in early motherhood (36), irrespective of anxiety. Furthermore, general measures omit items relevant to early motherhood, such as bonding, feeding, and sleeping (37).

The present study will firstly explore the relationships between prematurity and postpartum-specific anxiety using the subscales of the Postpartum Specific Anxiety Scale – Research Short Form (PSAS-RSF). Secondly, it aims to explore if postpartum-specific anxiety is differentially dependent on categories of prematurity. Finally, it aims to explore the relationship between postpartum-specific anxiety and stress in the NICU.

## 2 Materials and methods

### 2.1 Recruitment and procedure

Participants were recruited to an online study hosted on Qualtrics called ‘Gestational Age, Maternal Anxiety and Experiences of the NICU’, via social media platforms and word of mouth. The survey was released on two separate occasions (first: 14 February 2022–2 August 2022; second: 21 November 2022–12 March 2023). Screening questions ensured participants met eligibility criteria. Participants had to be ≥18 years old, English-speaking, and mothers of a live infant aged between birth and 12 months (corrected age for those that were premature infants, i.e., the infant’s chronological age minus their gestational age) born between 24 and 42 weeks’ gestation. Participants were additionally excluded from the study if their infant was currently in the NICU, or if they had not been discharged and well for at least three months via a screening question. There were no other specific exclusion criteria, to ensure a comprehensive demographic spread. All participants completed demographic questions and the PSAS-RSF. If they indicated that their infant had spent time in the NICU during the screening questions, they were asked for how many weeks their baby was in NICU in the demographics questionnaire, and additionally completed the Parental Stressor Scale: Neonatal Intensive Care Unit (PSS:NICU). Not all participants experienced the NICU, as we recruited mothers of infants with a gestational age between 24 and 42 weeks, hence including both mothers of term and mothers of premature infants. The screening question was asked to ensure participants who had not experienced the NICU were not asked questions about it. Participants could enter a prize draw to win a £25 voucher (or equivalent currency); e-mail addresses entered into the prize draw were separate from the dataset to ensure anonymity. The whole questionnaire took approximately 15 min to complete.

### 2.2 Measures

#### 2.2.1 Demographic questionnaire

Participants were asked a variety of questions, including their age, marital status, and occupation. Questions about the infant included their age and week of birth.

#### 2.2.2 Postpartum Specific Anxiety Scale – Research Short Form

The PSAS-RSF (38) is a 16-item short form of the PSAS (39). The scale focuses on the frequency of symptoms of maternal anxiety during the previous 7 days and is composed of four subscales with four items each: Maternal Competence and Attachment Anxieties includes items about parenting competence and the mother–infant relationship (McDonald’s ω = 0.78); Infant Safety and Welfare Anxieties encompasses anxieties around infant illness and accidental harm (McDonald’s ω = 0.89); Practical Infant Care Anxieties contains items about feeding and routine care (McDonald’s ω = 0.81); Psychosocial Adjustment to Motherhood includes anxieties regarding change to social, financial, and personal circumstances since the birth of the baby (McDonald’s ω = 0.70), each with four items, scored on a Likert scale (1 = not at all, 4 = almost always). The scale has acceptable reliability in the current study (McDonald’s ω*_h_* = 0.60), so the subscales were used in all analyses.

#### 2.2.3 Parental Stressor Scale: Neonatal Intensive Care Unit

The PSS:NICU (40) is one of the only scales measuring parental stress in relation to the NICU. The scale comprises 26 items with three subscales, which can be scored separately (Sights and Sounds of the Environment; Infant’s Appearance; Parental Role Alteration). Participants score on a Likert scale (1 = not at all stressful, 5 = extremely stressful), with all items featuring a ‘not applicable’ response. The measure was scored according to the ‘Stress Occurrence Level’, whereby only items experienced and rated by the participant are given a score (i.e., ratings of N/A receive a score of 0). The scale has good reliability in the current study (McDonald’s ω*_h_=* 0.70), as did both subscales: Sights and Sounds and Infant’s Appearance (McDonald’s ω = 0.93); Parental Role Alteration (McDonald’s ω = 0.93).

### 2.3 Statistical analyses

Analyses were conducted in R v4.3.1. Structural equation modeling (SEM) was used to analyze the relationship between gestational age (week of birth) and the subscales of the PSAS-RSF. Diagonally weighted least squares estimation was used as responses on the PSAS-RSF are ordinal on a one to four scale (41), with gestational age (week of birth) as the predictor variable, and the subscales of the PSAS-RSF as the outcome variable. Several demographic variables, including maternal age (continuous), ethnicity (0 = white, 1 = Black, Asian, or Minority Ethnic), educational attainment (ordinal with 1 representing higher attainment and 5 representing lower attainment), and current clinical diagnosis of anxiety (0 = yes, 1 = no), were also added to the model. As the subscales of the PSAS-RSF are latent variables in the model, confirmatory factor analyses were first conducted to ensure a good model fit. We utilized several methods for assessing fit; firstly, a normed chi square value (χ^2^/df) of between 1 and 2 indicates a good model fit (42), although it can be argued that values up to 5 are considered good (43). The standardized root mean square residual (SRMR) should have a value of <0.08 for good fit (44). Both the Comparative Fit Index (CFI) and Tucker–Lewis Index (TLI) should have values of >0.90 for acceptable fit and >0.95 for a good model fit (44). Finally, the root mean square error of approximation (RMSEA) should be below 0.08 for acceptable fit (45). The same values were also applied to the structural model in determining fit.

Following this, if there was a significant association(s) between PSAS-RSF subscales and gestational age in the SEM, then additional analysis on this would be conducted. Gestational age (week of birth) was split into the categories of prematurity as outlined by the WHO (3); extremely premature (<28 weeks gestation), very premature (28–<32 weeks gestation), late premature (32–<37 weeks gestation), and term (37+ weeks gestation) were used as a factor in a univariate ANOVA with the relevant PSAS-RSF subscales as the dependent variables (Holm *post-hoc* comparisons were performed where appropriate).

The relationship between the PSS:NICU and the PSAS-RSF was analyzed using a hierarchical regression analysis. Only participants who fully completed both the PSAS-RSF and PSS:NICU were included in this analysis.

### 2.4 Ethics statement

Ethical approvals were granted by the University of Liverpool Institute of Population Health Research Ethics Committee (ref:-IPH10606; 28 Jan 2022). Participants provided informed consent and were fully debriefed at the end of the study.

## 3 Results

### 3.1 Demographics

Of the 415 participants who responded to the survey, 178 (42.9%) were removed from the analysis due to non-completion. Of these 178, only 26 (14.6%) provided some response beyond the screening and consent questions. A comparison of these responses with the included sample can be found in **Supplementary Tables 1** and **2**; however, there were no significant differences between the groups. The final sample therefore consisted of 237 mothers who were predominantly married (54.4%), White (91.6%), and from the United Kingdom and the Republic of Ireland (64.4%). Approximately one-third of participants were in professional occupations (32.9%) and had completed an undergraduate education or equivalent (33.3%). Most participants indicated their infant had spent time in the NICU (67.5%); between 1 and 4 weeks for more than half (55.6%). See **Table 1** for full demographic characteristics.

**Table 1:** Maternal and infant demographic characteristics (N=237).

Maternal Characteristic	Value
Maternal age in years (mean ± SD)	30.79 ± 5.56
	
Country of Residence (N/%)	
UK and the Republic of Ireland	152 (64.4)
United States	54 (22.8)
Canada	10 (4.2)
Other	21 (8.9)
Ethnic Origin (N/%)	
White	217 (91.6)
Pakistani	2 (0.8)
Black African/Black Caribbean	7 (2.9)
Other	11 (4.6)
	
Marital Status (N/%)	
Married	129 (54.4)
Living with Partner	85 (35.9)
Separated	3 (1.3)
Single	20 (8.4)
	
Occupation (N/%)	
Managers, Directors, and Senior Officials	19 (8.0)
Professional Occupations	78 (32.9)
Associative Professional and Technical Occupations	3 (1.3)
Administrative and Secretarial Occupations	14 (5.9)
Skilled Trades Occupations	5 (2.1)
Caring, Leisure, and Other Service Occupations	28 (11.8)
Sales and Customer Service Occupations	21 (8.9)
Elementary Occupations	18 (7.6)
Not in a Paid Occupation	51 (21.5)
	
Educational Attainment (N/%)	
Completed postgraduate education (Master’s degree/PhD or equivalent)	54 (22.8)
Completed undergraduate education (degree or equivalent)	79 (33.3)
Completed A-Levels (or equivalent)	45 (19.0)
Completed GCSE’s (or equivalent)	35 (14.8)
No qualifications completed	9 (3.8)
Other	15 (6.3)
	
Housing Situation (N/%)	
Own home	130 (54.9)
Rent privately	66 (27.8)
Rent from the Local Authority	26 (11.0)
Live with Parents	8 (3.4)
Other	7 (3.0)
	
Occupant Number including participant (N/%)	
Two people	14 (5.9)
Three people	114 (48.1)
Four people	69 (29.1)
Five people	23 (9.7)
Six people or above	17 (7.2)
	
Current Clinical Diagnosis of Anxiety (N/%)	
Yes	112 (47.3)
No	120 (50.6)
Prefer Not to Say/Declined to Answer	5 (2.1)
	
Timing of Diagnosisb (N/%)	
Before Pregnancy	62 (26.2)
During Pregnancy	9 (3.8)
Postpartum	41 (17.3)
	
Prescribed Medicationb (N/%)	
Yes	63 (26.6)
No	47 (19.8)
Prefer Not to Say/Declined to Answer	2 (0.8)
	
Current Clinical Diagnosis of Depression (N/%)	
Yes	84 (35.4)
No	151 (63.7)
Prefer Not to Say/Declined to Answer	2 (0.8)
	
Timing of Diagnosisb (N/%)	
Before Pregnancy	47 (19.8)
During Pregnancy	7 (3.0)
Postpartum	30 (12.7)
	
Prescribed Medicationb (N/%)	
Yes	48 (20.3)
No	35 (14.8)
Prefer Not to Say/Declined to Answer	1 (0.4)
	
Infant Characteristic	Value
Infant age in weeks (mean ± SD)a	24.11 ± 13.69
	
Multiple Birth (N/%)	
Yes	16 (6.8)
No	221 (93.2)
	
Birth Order	
First	144 (60.8)
Second	59 (24.9)
Third	19 (8.0)
Fourth	10 (4.2)
Fifth	3 (1.3)
Sixth or after	2 (0.8)
	
Week of Birth (N/%)	
Extremely Premature (24-<28 weeks)	22 (9.28)
Very Premature (28-<32 weeks)	46 (19.40)
Late Premature (32-<37 weeks)	77 (32.5)
Term (37-42+ weeks)	92 (38.8)
	
Infant in NICU	
Yes	160 (67.5)
No	77 (32.5)
	
NICU Durationc (N/%)	
<1 week	3 (1.3)
1 to 4 weeks	89 (55.6)
5 to 10 weeks	45 (28.1)
11 to 15 weeks	17 (10.6)
16 or more weeks	6 (3.8)

^a^If participants indicated that their infant was born prematurely, infant age in weeks refers to corrected age at the time of survey completion

^b^Only participants who answered yes to clinical diagnosis included

^c^Only participants who indicated their child had spent time in the NICU included

### 3.2 Structural equation model

#### 3.2.1 Latent variables

##### 3.2.1.1 Maternal competence and attachment anxieties

The model was an excellent fit χ^2^/df = 0.14, CFI = 1.00, TLI = 1.03, RMSEA < 0.01, SRMR = 0.01.

##### 3.2.1.2 Practical infant care anxieties

The initial model represented a poor-to-adequate fit χ^2^/df = 6.29, CFI = 0.96, TLI = 0.87, RMSEA = 0.15, SRMR = 0.08. One pair of residuals were allowed to correlate, and this model represented an excellent fit for the data χ^2^/df = 0.12, CFI = 1.00, TLI = 1.02, RMSEA < 0.01, SRMR = 0.01.

##### 3.2.1.3 Infant safety and welfare anxieties

The model was an adequate-to-good fit for the data χ^2^/df = 3.69, CFI = 0.99, TLI = 0.96, RMSEA = 0.11, SRMR = 0.06.

##### 3.2.1.4 Psychosocial adjustment to motherhood

The model represented an excellent fit for the data χ^2^/df = 0.17, CFI = 1.00, TLI = 1.03, RMSEA < 0.01, SRMR = 0.01.

#### 3.2.2 Relationship between gestational age and postpartum specific anxiety

The overall model was an excellent fit for the data χ^2^/df = 1.17, CFI = 0.99, TLI = 0.99, RMSEA = 0.03, SRMR = 0.07. There was no significant association between maternal ethnicity (*B* = −3.24, SE = 1.85, *p =* 0.080, 95% CIs = −6.861 to −0.387), maternal age (*B* = −0.05, SE = 0.08, *p =* 0.528, 95% CIs = −0.173 to 0.088), or education (*B* = 0.02, SE = 0.35, *p =* 0.967, 95% CIs = −0.669 to 0.698), and gestational age at birth.

There was a significant, negative association between gestational age and Practical Infant Care Anxieties (*B* = −0.05, SE = 0.01, *p <* 0.001, 95% CIs = −0.012 to −0.052), Maternal Competence and Attachment Anxieties (*B* = −0.16, SE = 0.01, *p =* 0.005, 95% CIs = −0.024 to −0.004), and Infant Safety and Welfare Anxieties (*B* = −0.03, SE = 0.01, *p =* 0.020, 95% CIs = −0.026 to 0.002). There was no significant association between Psychosocial Adjustment to Motherhood (*B* = −0.02, SE = 0.01, *p =* 0.093, 95% CIs = −0.027 to 0.002) and gestational age.

**Figure 1:**
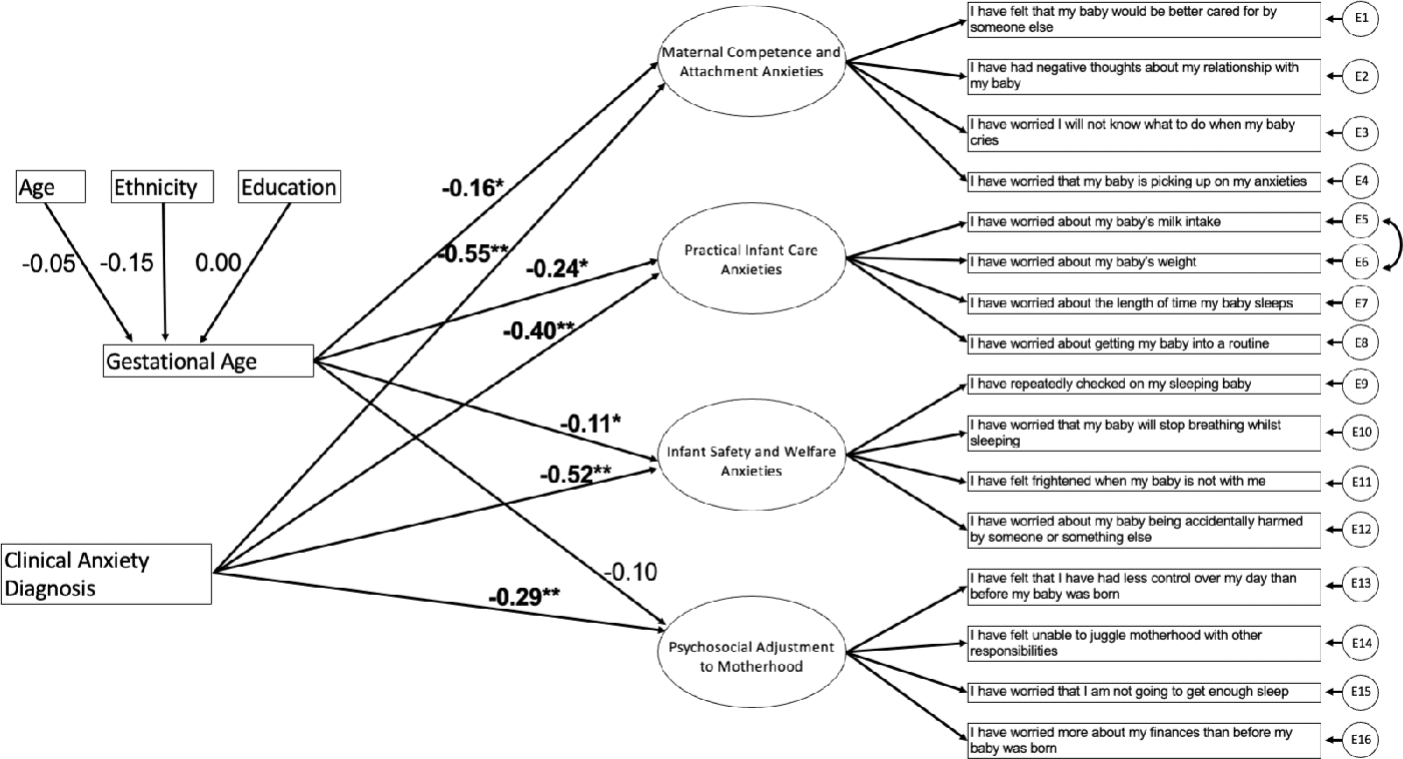
Structural Equation Model of the Relationship Between Gestational Age and the Sub-scales of the PSAS-RSF. Values represent standardised regression coefficients. *p<.05 **p<.001.

Current, clinical diagnosis of anxiety was significantly, negatively associated with all subscales of the PSAS-RSF, with those having a current clinical diagnosis of anxiety having significantly higher levels of anxiety (Maternal Competence and Attachment Anxieties *B* = −1.10, SE = 0.05, *p <* 0.001, 95% CIs = −0.538 to −0.343; Practical Infant Care Anxieties *B* = −0.79, SE = 0.05, *p <* 0.001, 95% CIs = −0.456 to −0.246; Infant Safety and Welfare Anxieties *B* = −1.03, SE = 0.06, *p <* 0.001, 95% CIs = −0.693 to −0.470; Psychosocial Adjustment to Motherhood *B* = −0.57, SE = 0.07, *p <* 0.001, 95% CIs = −0.464 to −0.205). See **Figure 1** for the full structural model.

### 3.3 Relationship between PSAS-RSF subscales and gestational age categories

Only subscales of the PSAS significantly associated with week of birth in the SEM analysis were explored in these analyses. Gestational age was split into the categories of gestational age outlined by the WHO (3), as above.

#### 3.3.1 Practical infant care anxieties

**Figure 2:**
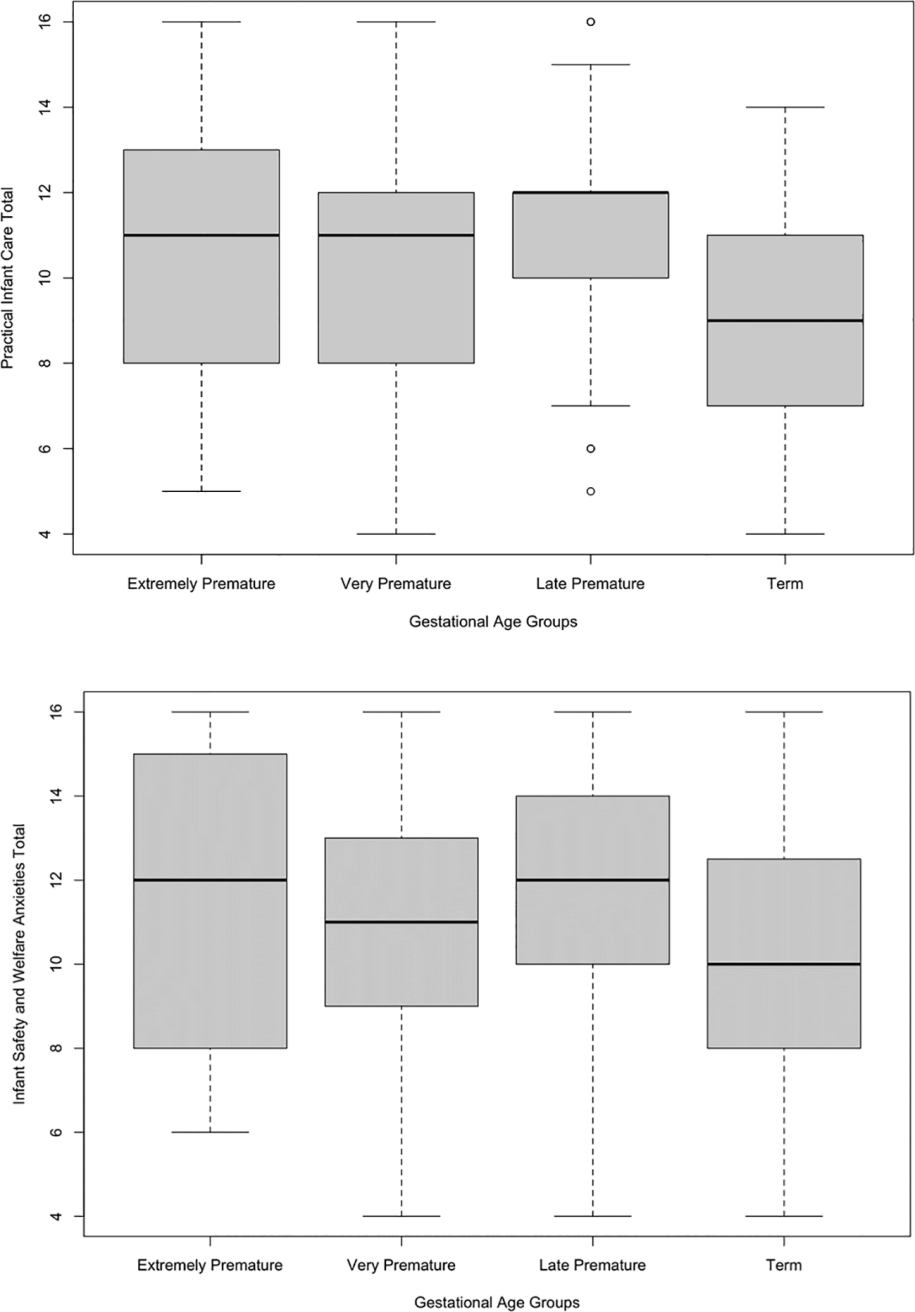
Boxplots of the relationship between Practical Infant Care Anxieties and Gestational Age Categories, and Infant Safety and Welfare Anxieties and Gestational Age Categories.

The overall model was significant, *F*(3, 233) = 8.00, *p <* 0.001, η^2 ^= 0.09. Holm corrected *post-hoc* tests demonstrated a non-significant difference between the extremely premature and very premature group (*p =* 0.722), late premature group (*p =* 0.567), and the term group (*p =* 0.162). There was also a non-significant difference between the late premature and very premature (*p =* 0.180), and term group (*p =* 0.145). There was, however, a significant difference between the late premature (EMM = 11.23, SE = 0.32) and term group (*p <* 0.001; EMM = 9.12, SE = 0.29); see **Figure 2**.

#### 3.3.2 Maternal competence and attachment anxieties

The overall model was not significant, *F*(3, 233) = 1.30, *p =* 0.275, η^2^= 0.02.

#### 3.3.3 Infant safety and welfare anxieties

The overall model was significant, *F*(3, 233) = 3.11, *p =* 0.027, η^2 ^= 0.04. Holm corrected *post-hoc* tests demonstrated a non-significant difference between the extremely premature and very premature group (*p* = 1.00), late premature group (*p* = 1.00), and the term group (*p =* 0.692). There was also a non-significant difference between the very premature and late premature group (*p =* 0.708), and term group (*p =* 0.745). There was a significant difference between the late premature (EMM = 11.80, SE = 0.36) and term group (*p =* 0.019; EMM = 10.40, SE = 0.33); see **Figure 2**.

### 3.4 Relationship between PSAS-RSF and PSS:NICU scores

A hierarchical regression was conducted to analyze the relationship between scores on the PSAS-RSF and PSS:NICU scores. Potential confounders (maternal age in years, infant age in weeks, and NICU duration in weeks) were decided *a priori* and added into Step 1, and all four PSAS-RSF subscales were added into Step 2.

The overall model was significant and predicted approximately 40% of variance in PSS:NICU scores adjusted *R* ^2^ = 0.40, *F*(4, 138) = 22.167, *p <* 0.001. *A* longer duration in the NICU was associated with higher PSS:NICU scores *B* = 0.35, SE = 0.14, *p =* 0.015, 95% CIs = 0.071 to 0.637. Higher PSS:NICU scores were associated with higher Infant Safety and Welfare Anxieties scores, *B* = 3.33, SE = 0.69, *p <* 0.001, 95% CIs = 1.970 to 4.690; see **Table 2**.

**Table 2:** Hierarchical regression models predicting PSS : NICU scores.

	Cumulative		Simultaneous		
	*R* ^2^ change	*F* change (df)	*B* (SE)	95% CIs	*p*
**Step 1**	0.06	2.95 (3, 142)*			
**Maternal Age**			−0.07 (0.31)	−0.682 to 0.528	0.802
**Infant Age**			0.06 (0.12)	−0.175 to 0.288	0.632
**NICU Duration**			0.35 (0.14)	0.071 to 0.637	0.015
**Step 2**	0.37	22.17 (4, 142)**			
**Maternal Competence and Attachment Anxieties**			0.80 (0.81)	−0.803 to 2.401	0.326
**Practical Infant Care Anxieties**			0.66 (0.75)	−0.825 to 2.146	0.381
**Infant Safety and Welfare Anxieties**			3.33 (0.69)	1.970 to 4.690	<0.001
**Psychosocial Adjustment to Motherhood Anxieties**			1.31 (0.74)	−0.164 to 2.781	0.081

*p < 0.05, **p < 0.01.

## 4 Discussion

### 4.1 Main findings

The aim of this study was to investigate the relationship between gestational age and symptoms of postpartum-specific anxiety, and whether this differed in terms of categories of prematurity. It also aimed to explore the relationship between postpartum specific anxiety and stress in the NICU. Gestational age had a significant negative association with all subscales, excluding Psychosocial Adjustment to Motherhood. There was only a significant difference between mothers of late premature and term infants in terms of Practical Infant Care and Infant Safety and Welfare Anxieties but there were no significant between-group differences in respect to Maternal Competence and Attachment Anxieties. Finally, there was a significant association between Infant Safety and Welfare Anxieties and stress scores after controlling for confounding variables, while those whose infants had spent a longer duration in NICU had significantly higher stress scores.

### 4.2 Interpretation of findings and comparison with published literature

It is widely accepted that premature birth increases the likelihood of developing symptoms of PPA (14)—specifically practical infant care, safety concerns, and competence anxieties as shown by the results of this study. One mechanism which may increase symptoms of PPA in mothers of premature infants is the reduced opportunity for bonding between mother and infant, resulting from maternal-infant separation which can leave mother’s feeling unconnected from their infant (46). This is prevalent when an infant is admitted to the NICU (47). Maternal–infant separation can occur from the point of birth, particularly if the infant has medical difficulties (48), the consequences of which can be linked to parental role alteration anxieties (49). The benefits of bonding are well documented in mothers of term and premature infants (50), the absence of which due to separation can increase symptoms of PPA (51).

The results of this study demonstrated that mothers of later-premature infants had significantly higher practical infant care anxieties compared to the other categories of prematurity that encompass concerns over sleep and infant routine, as well as anxieties surrounding feeding. Mothers of later-premature infants may have low perceptions of their caregiving ability immediately upon hospital discharge, perhaps due to additional care needs relating to the infant (52). Furthermore, infant feeding as a source of anxiety and stress for mothers is well-established, particularly in NICU (20). Qualitative studies further reflect this, with one study emphasizing maternal concerns about their parental ability, with many expressing low confidence in their caregiving abilities as a result of additional care needs (53). Furthermore, these increased anxieties may be due to constraints of routines during hospital admission (54), the consequences of which persist across the first year postpartum. Concerns regarding infant safety may be commonplace in mothers of premature infants due to medical complications resulting from birth, which may leave mothers feeling anxious about their baby’s survival (55), and hypervigilant surrounding discharge (11); this may be more prevalent in mothers of later premature infants as they transition home, particularly regarding the perceived fragility of their infant in comparison to other babies (56).

However, the results demonstrated no significant between-group differences for Maternal Competence and Attachment Anxieties, suggesting that anxieties are similar across gestational groups. While these findings differ from previous literature, the SEM model controlled for clinical diagnoses of anxiety as well as known demographic factors such as age and ethnicity that are known to have an influence on levels of anxiety after birth. However, these demographic factors were not controlled for in the ANOVA, so it may be that they have a more pertinent influence on anxieties surrounding competence. Furthermore, there were only no significant differences between Maternal Competence and Attachment anxieties, whereas Infant Safety and Welfare and Practical Infant Care Anxieties did significantly differ between the groups. It may be that common anxieties after preterm birth are infant-focused, rather than anxieties surrounding competence and parental efficacy.

The results of this study showed a significant association with PSS:NICU and PSAS-RSF scores, suggesting that elevated levels of stress in the NICU are related to high levels of anxiety. While anxiety and stress differ conceptually, they share some overlap in symptoms (57), and general measures of stress and anxiety contain some similar items. However, the PSS:NICU is setting-specific, and measures distinct stressors in the NICU environment such as Sights and Sounds, Infant Appearance, and Parental Role Alterations while in the unit. This differs from the PSAS-RSF, which measures anxieties relating to all aspects of motherhood, and is not specific to a setting. Therefore, our findings demonstrate that stress as a result of the NICU is associated with maternal- and infant-focused anxieties across the first year of life.

When considering infant appearance in the NICU, approximately 99% of mothers in one study reported this caused them significant stress (58). This may include concerns such as cuts and bruises, and the small size of the infant (59). Furthermore, parental role alteration anxieties are common in mothers of premature infants, and it appears that this is a significant source of stress. Qualitative studies have revealed mothers feel unprepared with motherhood and the NICU routine (60). The extent to which a mother adapts well to their new parental role may also be dependent on other factors—increased engagement from staff during NICU hospitalization reduces parental role alteration anxieties (61). A recent study indicates that mothers may be anxious about their baby’s long-term development following NICU discharge due to potentially complex medical needs (62), which may link to Infant Safety and Welfare Anxieties. Significant sources of stress, such as those most prevalent in the NICU, have been associated with symptoms of state and trait anxiety (20).

### 4.3 Strengths, limitations, and future directions

This is one of few quantitative studies to consider postpartum-specific anxiety within the context of categories of gestational age, allowing for targeted interventions for mothers of premature infants. It is also used the PSS:NICU retrospectively, which allowed the consideration of the sustained effect of having an infant in the NICU over the first postpartum year. This is the first study to consider PPA using a context-specific measure within the NICU environment. While online data collection allowed for a large number of participants to take part quickly and easily, attrition rates can be high (42.9% in this study). Furthermore, much of the population were highly educated professionals and of White ethnicity. Future studies should make concerted efforts to recruit from diverse socio-economic and ethnic backgrounds, particularly as those of a low socio-economic status are known to experience higher rates of anxiety following a premature birth (63). Given the results of this study regarding the subscales of the PSAS-RSF and categories of gestational age, future studies may wish to undertake a qualitative approach, to further explore the experiences of the postpartum period for mothers of preterm infants, particularly with regard to the anxieties they might face in the NICU, after discharge, and throughout the first postpartum year. Future research should also consider the implementation of specific interventions relating to anxieties shown in the subscales of the PSAS-RSF, which may potentially reduce the adverse effects resulting from a premature birth.

### 4.4 Clinical implications

This study further highlights the need for differential support both during the NICU and across the first postpartum year for mothers of premature infants. Interventions addressing anxiety and stress during NICU admission which focus on structured nursing interventions have been successful at reducing anxiety and stress (64), but interventions must now focus, in the first instance, on addressing concerns specific to the postpartum period, and this support must be differential dependent on categories of gestational age. For example, as the results of this study indicated, mothers of later-premature infants score significantly higher on the practical infant care anxieties subscale, and so interventions may wish to target concerns surrounding this, such as feeding and washing. This, however, may not be as applicable to other categories of prematurity. Increasingly, there have been attempts in the United Kingdom to implement Family Integrated Care (FiCare) in neonatal units by working to promote relationships between staff and parents and encouraging education to increase wellbeing and involvement in infant care (65). Recent research demonstrates that implementation of FiCare outside of the UK has been successful at reducing parental anxieties during infant admission (66), and parents report the experience to improve feelings of self-efficacy and parental role adjustment (67) which is important to consider when considering the results of this study. However, recent calls for initiatives such as FiCare to extend post-discharge (68) are warranted given the sustained impact of preterm birth and NICU admission across the first postpartum year, as demonstrated in this study. The current proposed approach to the widespread implementation of FiCare in the United Kingdom is inconsistent (69) and requires careful consideration given the time commitments involved for both parents and clinicians, and the limited evidence as to its effectiveness at reducing anxiety long-term (70). Similar family-centered resources that promote the mother**–**infant relationship, such as the Newborn Behavioral Observation (NBO) system have been recommended for use in the UK (71), and have been shown to promote maternal knowledge and empowerment regarding their baby, particularly when born preterm (72).

### 4.5 Conclusion

This study investigated the effect of prematurity on anxiety and stress in the NICU, and is one of the few to do this using a postpartum-specific measure of anxiety. Results showed that week of gestation was significantly associated with all PSAS-RSF subscales excluding Psychosocial Adjustment to Motherhood Anxieties. Mothers of later premature infants have higher anxieties surrounding infant safety and competence than other categories. Furthermore, postpartum-specific anxiety was positively associated with stress in the NICU. The findings of this study are largely in support of previous literature. Future research must now further consider the sustained impact of a NICU admission on rates of anxiety in mothers, using a qualitative approach that specifically targets the subscales of the PSAS-RSF, as this study demonstrates that they are experienced differentially dependent on gestational age.

## Data availability statement

The raw data supporting the conclusions of this article will be made available by the authors, without undue reservation.

## Ethics statement

The studies involving humans were approved by the University of Liverpool Institute of Population Health Research Ethics Committee (ref:-IPH10606; 28 Jan 2022). The studies were conducted in accordance with the local legislation and institutional requirements. The participants provided their written informed consent to participate in this study.

## Author contributions

SW: Conceptualization, Data curation, Formal Analysis, Investigation, Methodology, Project administration, Resources, Software, Visualization, Writing – original draft. PC: Supervision, Validation, Writing – review & editing. AK: Supervision, Validation, Writing – review & editing. SS: Supervision, Validation, Writing – review & editing. VF: Conceptualization, Investigation, Methodology, Resources, Supervision, Validation, Writing – review & editing.
