# Supplementary material for: Associations between prematurity, postpartum anxiety, neonatal intensive care unit admission, and stress
Source: Front Psychiatry. 2024 Feb 23;15:1323773. doi: 10.3389/fpsyt.2024.1323773 (PMC10921229; doi:10.3389/fpsyt.2024.1323773)
Supplement: Supplementary file 1 [file Table_1.docx]

| **Supplementary Table 1.** Paired Samples t-test between excluded and included participants for continuous variables (N values listed for each variable). | | | |
| --- | --- | --- | --- |
| **Variable** | **Included**  **N/(Mean(SD))** | **Excluded N/(Mean(SD))** | ***t*(df), p-value, *d*** |
| **Maternal Age (weeks)** | N=237  30.79(5.56) | N=26  30.81(9.01) | ^a^t=0.01(27.13), p=.994, *d*=.002 |
| **Infant Age (weeks** | N=237  24.11(13.69) | N=13  27.69 (17.13) | t=0.91(248), p=.366, *d*=.258 |
| **Gestational Age** | N=237  34.29(4.54) | N=13  32.38 (4.77) | t=-1.47(248), p=.142, *d*=-.419 |
| **Occupant Number (including participant)** | N=237  2.68(1.09) | N=23  2.87(1.77) | ^a^t=0.51(23.66), p=.617, *d*=.163 |
| **NICU Duration** | N=160  6.22(11.22) | N=9  5.89(4.23) | t=-.088(167), p=.930, *d*=-.030 |
| ^a^Welch’s t-test as equal variances not assumed  NB: Of the 178 participants that were excluded from the survey, only 26 (14.61%) provided some response beyond the screening and consent questions, which are analysed here. N differs as participants withdrew from the study at different points. | | | |
