# Supplementary material for: Associations between prematurity, postpartum anxiety, neonatal intensive care unit admission, and stress
Source: Front Psychiatry. 2024 Feb 23;15:1323773. doi: 10.3389/fpsyt.2024.1323773 (PMC10921229; doi:10.3389/fpsyt.2024.1323773)
Supplement: Supplementary file 2 [file Table_2.docx]

| **Supplementary Table 2.** Mann-Whitney U test conducted between groups (included/excluded) for ordinal variables (N values listed for each variable). | | | |
| --- | --- | --- | --- |
| **Variable** | **Included N/Median(Range)** | **Excluded N/Median(Range)** | **W, p-value, A** |
| **Occupation** | N=237  Median=4(9) | N=23  Median=4(9) | W=2773, p=.889, A=.963 |
| **Birth Order** | N=237  Median=1(2) | N=13  Median=1(2) | W=1318.50, p=0.314, A=0.71 |
| **Educational Attainment^a^** | N=222  Median=2(4) | N=21  Median=1(5) | W=2459.50, p=.666, A=.891 |
| ^a^ “Other” option excluded from this analysis  NB: Of the 178 participants that were excluded from the survey, only 26 (14.61%) provided some response beyond the screening and consent questions, which are analysed here. N differs as participants withdrew from the study at different points. | | | |
